# Molecular Action of Hydroxytyrosol in Attenuation of Intimal Hyperplasia: A Scoping Review

**DOI:** 10.3389/fphar.2021.663266

**Published:** 2021-05-21

**Authors:** Ubashini Vijakumaran, Muhammad Dain Yazid, Ruszymah Bt Hj Idrus, Mohd Ramzisham Abdul Rahman, Nadiah Sulaiman

**Affiliations:** ^1^ Centre for Tissue Engineering and Regenerative Medicine, Universiti Kebangsaan Malaysia Medical Centre, Kuala Lumpur, Malaysia; ^2^ Department of Physiology, Faculty of Medicine, Universiti Kebangsaan Malaysia Medical Centre, Kuala Lumpur, Malaysia; ^3^ Department of Surgery, Faculty of Medicine, Universiti Kebangsaan Malaysia Medical Centre, Kuala Lumpur, Malaysia

**Keywords:** hydroxytyrosol, intimal hyperplasia, neointimal hyperplasia, smooth muscle cell, endothelial cell

## Abstract

**Objective:** Hydroxytyrosol (HT), a polyphenol of olive plant is well known for its antioxidant, anti-inflammatory and anti-atherogenic properties. The aim of this systematic search is to highlight the scientific evidence evaluating molecular efficiency of HT in halting the progression of intimal hyperplasia (IH), which is a clinical condition arises from endothelial inflammation.

**Methods:** A systematic search was performed through PubMed, Web of Science and Scopus, based on pre-set keywords which are Hydroxytyrosol OR 3,4-dihydroxyphenylethanol, AND Intimal hyperplasia OR Neointimal hyperplasia OR Endothelial OR Smooth muscles. Eighteen *in vitro* and three *in vitro* and *in vivo* studies were selected based on a pre-set inclusion and exclusion criteria.

**Results:** Based on evidence gathered, HT was found to upregulate PI3K/AKT/mTOR pathways and supresses inflammatory factors and mediators such as IL-1β, IL-6, E-selectin, P-selectin, VCAM-1, and ICAM-1 in endothelial vascularization and functioning. Two studies revealed HT disrupted vascular smooth muscle cells (SMC) cell cycle by dephosphorylating ERK1/2 and AKT pathways. Therefore, HT was proven to promote endothelization and inhibit vascular SMCs migration thus hampering IH development. However, none of these studies described the effect of HT collectively in both vascular endothelial cells (EC) and SMCs in IH *ex vivo* model.

**Conclusions:** Evidence from this concise review provides an insight on HT regulation of molecular pathways in reendothelization and inhibition of VSMCs migration. Henceforth, we propose effect of HT on IH prevention could be further elucidated through *in vivo* and *ex vivo* model.

## Introduction

### Intimal Hyperplasia and Current Treatments

Annually, millions of coronary artery bypass surgery (CABG) and percutaneous coronary interventions (PCI) are performed to treat ischemic heart disease. However, the development of intimal hyperplasia (IH) limits the long-term efficacy of these cardiovascular interventions (Mylonaki et al., 2018). Intimal hyperplasia is defined by thickening of the intimal layer of a blood vessel as a response to endothelial injury, which occurs during or post-surgical procedures such as PCI or CABG (Gellman et al., 1991). Endothelial injury triggers inflammation and platelet activation which subsequently stimulates the proliferation and migration of smooth muscle cells (SMCs) from media toward the intimal layer. SMCs migration is highly assisted by the secretion of inflammatory factors and mediators and degradation of multiple extracellular matrix (ECM) components in the media and adventitia (Jennette and Stone, 2014). This cascade reaction eventually leads to atherosclerosis where the blood vessel narrowed, and surrounding tissues falls into ischemic condition. Unfortunately, the formation of IH decreases the patency of bypass grafted veins to 40% after 10–20 years following surgery (de Vries and Quax, 2018).

Despite cutting edge therapies, IH remains as the main risk after CABG with no known remedy to reduce or relinquish the ever-progressing condition. Antithrombotic drugs are the classic approach to prevent IH (Hillis et al., 2012; Anderson et al., 2013). However, prolonged dual-antiplatelet therapy post angioplasty and stent implantation increases the risk of internal bleeding (Costa et al., 2015; Urban et al., 2019). First-generation drug-eluting stent (DES) incorporated with antiproliferative drugs like Sirolimus and Paclitaxel, were used to replace bare-metal stent (BMS) (Stone et al., 2007) has significantly reduced the recurrence of occlusion (Stettler et al., 2007; Jennette and Stone, 2014). Unfortunately, increased late stent thrombosis were also reported (Stone et al., 2007). DES efficiently prevent the migration of SMCs by disrupting SMCs cell cycle but with the price of delayed re-endothelization due to the antiproliferative effect of the drug on endothelial cells (ECs) (Camenzind et al., 2007; Joner et al., 2008).

Moving forward, bioresorbable stent (BRS) technology were introduced where the stents could be completely resolved after six months of implantation (Luo et al., 2014) leaving zero traces of stents material. This ultimately reduces future complications like stent migration, endothelial dysfunction, and restenosis (Gonzalo and Macaya, 2012). Unfortunately, BRS mechanical properties i.e. strut thickness, causes vessel injury and subsequently leads to platelet recruitment and thrombosis (Lee and Hernandez, 2018). In addition to that, concern about the degradation and disintegration of BRS into its by-products and its elimination in the coronary artery adds more challenges to the use of BRS. Large and small randomized trials of BRS implantation, unveiled thrombosis and intimal proliferation at one year follow up (Jinnouchi et al., 2019). Moreover, Optical Coherence Tomography (OCT) of an implanted Bioresorbable Novolimus-Eluting Coronary in patient revealed that the implanted scaffold collapsed and increased of neointimal proliferation in the artery (Alfonso and García-Guimaraes, 2017); Braun et al., 2016). Absorb Bioresorbable Vascular Scaffold (BVS; Abbott Vascular) is the first FDA-approved BRS, but it failed to ensure sustained success with increased late thrombosis events reported that leads to its withdrawal from the market due to low demand (Jinnouchi et al., 2019). BRSs are being redeveloped by taking into consideration several issues that include the strut thickness, degradation efficiency, scaffold thrombosis, and currently waiting to be evaluated in large-scale clinical trials (Regazzoli et al., 2017).

### Plant-Based Approach for IH

Various herbal plant-based components were studied for their cardiovascular protection effect (Barnard et al., 2019; Kim et al., 2019; Tuso et al., 2015). Xu et al. compiled a list of natural plant derived compounds such as flavonoids, polyphenols, alkaloids, and terpenes that were found to efficiently suppress VSMCs migration and proliferation (Xu et al., 2018). They further elucidated the involvement of typical cell regulatory and inflammatory pathways including MAPKs, PI3K/Akt, JAK-STAT, FAK, and NF-κB in VSMCs migration. However, they focused solely on activity of plant base compounds on VSMCs and not collectively with endothelial cells which is also an essential cell in pathophysiology of IH.

Polyphenol such as resveratrol is the most studied compound in IH prevention. Balloon catheters coated with resveratrol effectively deliver resveratrol to the targeted site and successfully reduce IH development in rabbit models (Tolva et al., 2016). In addition to that, a series of *in vivo* animal studies showed that resveratrol promoted re-endothelization and vascular healing post-surgical anastomosis (Yurdagul et al., 2014; Karaarslan et al., 2015; Kamann et al., 2019). Kamann et al. reported that resveratrol increases ECs proliferation via activating extracellular signal-regulated kinase (ERK) and estrogen receptor-dependent pathway under laminar shear stress (Yurdagul et al., 2014). Interestingly, curcumin also ameliorated IH by increasing endothelial angiogenesis and proliferation in an artery injured rat (Chen et al., 2015).

Alternatively, quercetin (Khandelwal et al., 2012) and salvianolic acid A (SAA) (Sun et al., 2012) were also found to inhibit proliferation of VSMCs too. Intriguingly, a green tea polyphenol, epigallocatechin-3-gallate (EGCg), suppressed neointimal hyperplasia (NIH) in rabbit model by inhibiting the proliferation of VSMCs via inactivation of MAPKs pathway In a recent study, Wei delivered mesoporous silica nanoparticles encapsulated honokiol (HNK), a small molecule polyphenol after balloon injury and HNK greatly suppressed intimal thickening by reducing phosphorylation of Smad3 (Wei et al., 2020).

### Hydroxytyrosol as an Innovative Approach

Olive oil is the primary source of fat and polyphenols in Mediterranean Diet (MD) (Widmer et al., 2015). In 2013, the United Nations Educational, Scientific and Cultural Organization (UNESCO) include MD in the “Representative List of the Intangible Cultural Heritage of Humanity”. MD was also classified in the 2015–2020 Dietary Guidelines for Americans as a healthy diet (Romagnolo and Selmin, 2017). Phytochemicals from olive plant showed positive correlation with the reduction of cardiovascular diseases symptoms and risk factors (Tejada et al., 2016; Guasch-ferré et al., 2019).

Hydroxytyrosol (HT) is a most potent antioxidant, with 154.16 g/mol M mass found in the olive plant (Granados-Principal et al., 2010). HT is naturally derived from the hydrolysis of oleuropein (Tagliafierro et al., 2015) and alternatively, from dopamine metabolism in humans (Rodríguez-Morató et al., 2016). In nature, HT is hydrophilic hence readily absorb in a dose-dependent manner in animals and humans and are excreted in the urine as glucuronide conjugates (Kamil et al., 2020). HT is a well-studied phytochemical for its vascular protection (Hernáez et al., 2017; Nemzer et al., 2019), antioxidant (Adawiyah Razali et al., 2019; Soler-Cantero et al., 2012; Tejada et al., 2016), anti-inflammatory (Chin and Pang, 2017; Ng et al., 2017; Vilaplana-Pérez et al., 2014; Li et al., 2017), anti-atherogenic properties including the inhibition of LDL oxidation (Storniolo et al., 2019); and anti-platelet aggregation (De Roos et al., 2011). A couple of independent research elucidated HT potential in the attenuation of IH development (Xu et al., 2018; Man et al., 2020) However, HT has not been employed in any *in vivo* model to treat IH. Therefore, we aim to collect the scientific evidence of HT in the suppression of IH. This systematic review collate *in vitro* and *in vivo* studies that elucidate the underlying molecular action of HT in the attenuation of IH.

## Methodology

### Search Strategy

**FIGURE 1:**
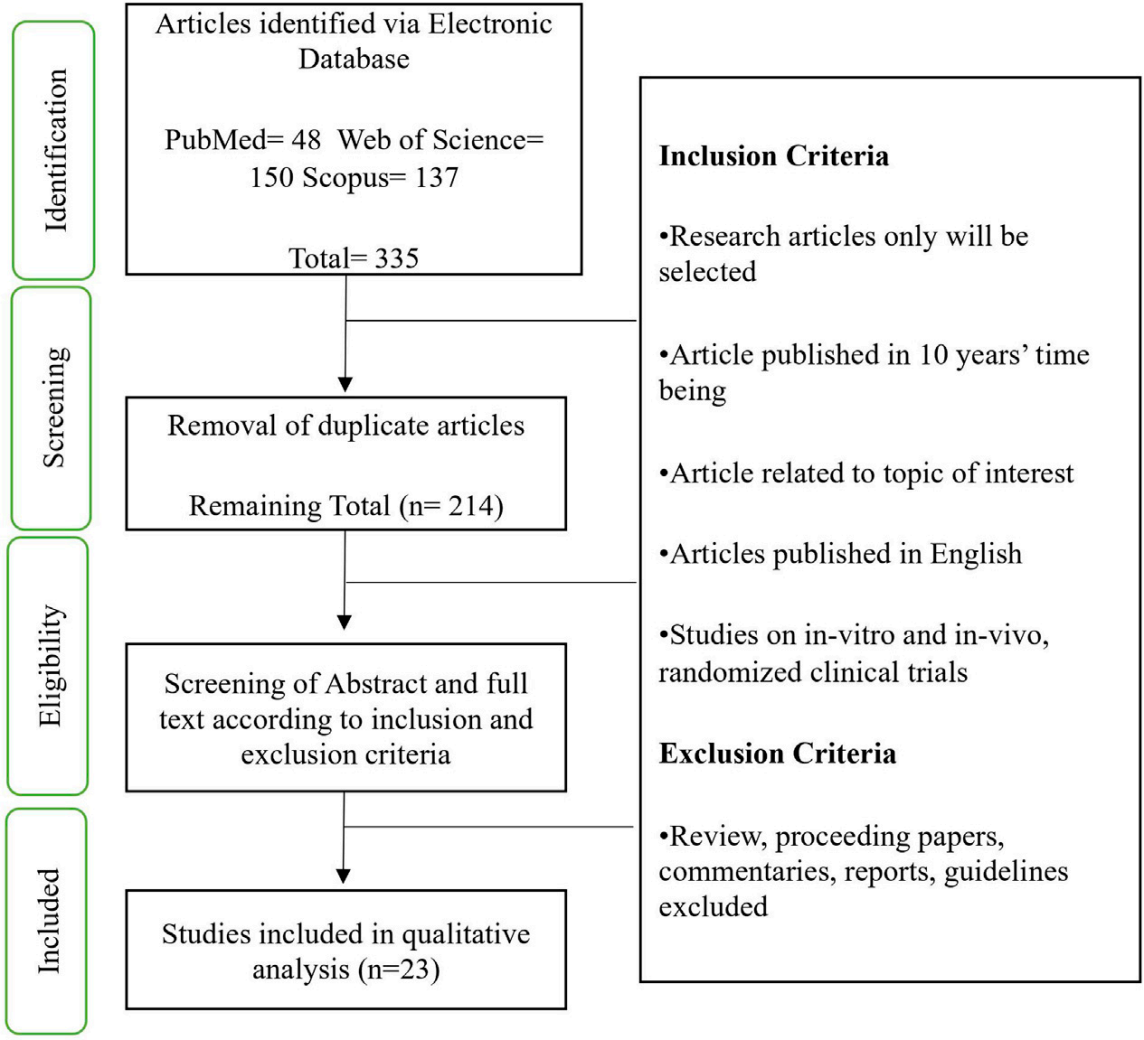
Flow chart represent selection and screening process based on PRISMA.

The selection and screening process were carried out based on PRISMA guideline as presented in Figure 1. A systematic screening through three databases (PubMed, Scopus and Web of Science) were performed. Original articles related to the molecular action of Hydroxytyrosol in intimal hyperplasia were searched using the following keywords: Hydroxytyrosol OR 3,4-dihydroxyphenylethanol AND Intimal hyperplasia OR Neointimal hyperplasia OR Endothelial OR Smooth muscle cells.

### Selection Criteria

Full-text articles published between 2011–2020 in English were included. Only research/original articles were selected while review articles, proceeding abstract, and case studies were excluded. The search included all *in vitro* and *in vivo* studies. Titles and abstracts were meticulously screened and only articles that correlate to the molecular and cellular mechanism of action of Hydroxytyrosol in intimal hyperplasia were selected.

### Data Extraction and Management

Two independent reviewers thoroughly screen the selected research articles. All related articles from the database searches were combined and duplicates were removed. The rest of the articles were screened further to meet the selection criteria. The title was first screened, follow by the abstracts for relevance to the selected topic. Unrelated articles that do not fall into the inclusion criteria were removed. The extracted data are tabulated concisely as follows: 1) Reference 2) Aim 3) Cells and Treatment 4) Test(s) 5) Finding(s) 6) Signaling molecules/Pathways 7) Conclusion/correlation with IH.

### Quality Evaluation

The quality of the selected studies was validated using a modified version of Office of Health Assessment and Translation (OHAT). The checklist is designed as presented in Table 3 to evaluate the potential risk of bias of both *in vivo* and *in vitro* studies by assessing 1) reporting bias, 2) performance bias, 3) detection bias, and 4) selection bias.

## Result

### Search Results

Initially, a total of 335 articles were identified from all database search and 216 articles remained after the removal of duplicates. The reviewers validate selected articles independently according to designed inclusion and exclusion criteria to minimize bias. Screening of title and abstract were done twice and a total of 35 papers were identified. During the final screening of the full text, 11 non-related articles, and 3 articles that used nonvascular cells were excluded. In the end, a total of 19 *in vitro* and 4 both *in vitro* and *in vivo* studies were selected for the review. Figure 1 shows the selection and screening process based on PRISMA guidelines.

### Study Characteristics

Three electronic database searches identified 19 *in vitro* (Table 1) and 4 studies that include both *in vivo* and *in vitro* (Table 2) analysis related to the action of HT in enhancing endothelial function and inhibiting proliferation of VMSCs which are involved in the suppression of intimal hyperplasia development. Data extracted from the selected articles is presented in Table 1. Most experiments were conducted utilizing human umbilical vein endothelial cells (HUVECs), human vascular endothelial cells (HVEC) and porcine pulmonary artery endothelial cells (PAECs). However, monocyte cell lines such as U937 and Jurkat were also used in 3 studies. Bovine vascular smooth muscle cells (BVSMVs) and human microvascular endothelial cells (HMVECs) were utilized in 2 studies. Human microvascular cell line, bovine aorta endothelial cells (BAECs), human peripheral blood cells, myoblast, rat vascular smooth muscle cells, and vascular adventitia fibroblast were also utilized. Two *in vivo* studies were conducted on mice while one was done in a rat model.

**TABLE 1:** *In vitro* studies outcomes.

No	References	Aim	Cells and treatment	Tests	Findings	Signaling molecules/Pathways	Conclusion/correlation with IH
1	Nakbi et al. (2011)	To investigate the potential of HT and T on oxidative damage caused by ROS production and MMP-9 expression in PMA induced THP-1	Cells THP-1 Treatment HT (1, 5, 10 and 50 μM) and T (0.05, 0.15, 0.5 and 2 mM) for 4, 15 and 24 h followed by addition of PMA (0.1 μM)	1. Superoxide anion production 2. MMP-9 expression	1. HT and T reduced superoxide release	ROS	HT reduced MMP-9 production that could prevent the migration of smooth muscle cell
2	Scoditti et al. (2012)	To study polyphenols effect on COX-2 and MMP-9 expression induced by pro-angiogenic factor PMA	Cells 1. HUVEC 2. HMEC-1 Treatment HT (0.1–50 µMmol/L)	1. Cell cytotoxicity 2. MMP-9 release 3. MMP-9 gelatinolytic activity 4. PGE2 production 5. COX-2, COX-1, b-actin, and p65 NF-kB expression 6. ROS production	1. HT prevented inflammatory tube formation and cell migration 2. HT inhibited MMP-9 expression 3. HT inhibited COX-2 activity 4. HT decreased ROS level 5. HT suppressed translocation and transactivation of p65 NF-κB	NF-κB	HT suppressed the ROS level and NF-κB activation that regulates the proliferation of endothelial and smooth muscle cells
3	Lamy et al. (2014)	To investigate effect of phenolic compounds toward endothelial cell angiogenesis	Cells 1. HUVECs (HMVECs-d-Ad) Treatment 50 μM olive oil compounds for 18 followed by addition of 1 μg/ml VEGF	1. Tube formation 2. Cell proliferation 3. Cell migration 4. VEGFR-2 phosphorylation study	1. HT suppressed VEGF-induced tube formation 2. HT inhibited cell proliferation 3. HT inhibited phosphorylation of VEGFR-2 4. HT suppressed phosphorylation of ERK-1/2 and SAPK/JNK	1. VEGF 2 2. ERK-1/2 3. SAPK/JNK	HT potently suppressed ERK-1/2, SAPK and JNK pathways involved in endothelial apoptosis
4	Scoditti et al. (2014)	To study the HT effect on MMP-9 expression involved in COX-2/PGE2 pathway in PMA stimulated human monocytes stimulated	Cells 1. PBMC 2. U937 Treatment HT (1–10 μmol/L) for 1 h followed by stimulation with 30 nmol/L PMA for 0–24 h	1. MMP-9 and TIMP-1 secretion 2. PGE2 production 3. COX-2, COX-1, PKCa, PKCb1, NF-kB expression 4. MMP-9, COX-2, MCP-1, ICAM-1, IL-1b, TNF-a gene expression 5. NF-kB activation 6. PKC translocation	1. HT suppressed MMP-9 secretion 2. HT reduced MMP-9 mRNA levels 3. HT suppressed PGE2 production 4. HT inactivated NF-kB 5. HT decreased MCP-1, ICAM-1, IL-1b, and TNF-α mRNA level 6. HT inactivated PKCα and PKCβ1	1. PGE2 2. NF-kB	HT exhibits protection against vascular endothelial inflammation by suppressing inflammatory cytokines and activating COX-2 and PGE2 pathway
5	Zrelli et al. (2011b)	To study the potential of HT on ROS reduction by enhancing catalase activity through AMPK-FOXO3a pathway	Cells PPAECs Treatment HT (10, 30 and 50 μM)	1. ROS production 2. Catalase mRNA level 3. Phosphorylation of AMPKα and AMPKβ1 4. Protein level of catalase, FOXO3a and AMPK	1. HT reduced ROS 2. HT increased catalase expression 3. HT upregulated FOXO3a expression and mediated nuclear translocation 4. HT activated AMPK phosphorylation	AMPK–FOXO3	HT positively regulated endothelial oxidative defense while prevents endothelial dysfunction and apoptosis by activating AMPK-FOXO3 pathways
6	Zrelli et al. (2013)	To study the effect of hydroxytyrosol with carbon monoxide-releasing Molecule-2 in prevention of endothelial dysfunction through NO production and NFκB inactivation	Cells PAECs Treatment HT (1, 10, or 100) μmol/L	1. eNOS,NFκBp65, IκBα, cleaved 2. caspase-3 expression 3. NO production 4. Cell cytotoxicity 5. Cell morphology 6. NFκB activation	1. HT inhibited cytotoxicity 2. HT suppressed cellular damage 3. HT inhibited apoptotic morphology changes and apoptotic cell death 4. HT alone and HT + CORM-2 reduced NFκBp65 protein level 5. HT + CORM-2 increased Enos phosphorylation 6. HT + CORM-2 increased NO release 7. HT + CORM-2 blocked activation of caspase-3 8. HT alone inhibited NFκBp65 phosphorylation while CORM-2 enhanced it 9. HT + CORM-2 inactivates NFκB	NFκB	HT + CORM-2 potentially inhibited endothelial apoptosis by inhibiting caspase 3 and NFκB pathway while supported vascular healing through NO production
7	Abe et al. (2012)	To examine the potential of olive oil phenols in inhibition of smooth muscle cell proliferation through a G1/S cell cycle block regulated by ERK1/2	Cells BVSMCs Treatment HT (1, 10, or 100 μmol/L)	1. Cell proliferation 2. Cell cycle 3. (ERK)1/2 phosphorylation	1. HT inhibited cell proliferation 2. HT disrupted cell cycle and controlled over proliferation 3. HT inhibited ERK1/2 phosphorylation	ERK1/2	HT has potential to inhibit intimal hyperplasia by reducing migration and proliferation of SMC via blocking cell cycle regulated by ERK1/2 phosphorylation
8	Torul et al. (2020)	To evaluate phenolic compounds of olive extract on endothelial toxicity induced by hydrogen peroxide	Cells HUVECs Treatment HT (1.0–10.0 μmol/L	1. Determination of phenolic compounds 2. Induction of ROS 3. Cell cytotoxicity	1. HT suppressed cell toxicity 2. HT decreased ROS production	ROS	HT shown to decrease ROS generation in endothelial which could promote vascular healing
9	Fortes et al. (2012)	To investigate effect of hydroxytyrosol and tyrosol in preventing inflammatory angiogenesis	Cells 1. HUVECs 2. HMECs 3. BAECs Treatment HT 10 mg/ml	1. Cell cytotoxicity 2. Cell migration 3. Tube formation 4. Cell cycle analysis 5. MMP-2 production	1. HT inhibited cell proliferation 2. HT inhibited cell migration 3. HT suppressed tube formation 4. HT enhances apoptosis 5. HT regulated cell cycle 6. HT inhibited MMP-2 activity		HT regulated endothelial cell cycle while decreased production of MMP-2 that possibly could prevent smooth muscle cells migration
10	Abate et al. (2020)	To investigate the effect of HT in endothelial vascularization	Cells 1. HUVECs 2. HVECs Treatment (0–160 µM) for 24 and 48 h	1. Cell viability 2. Cell proliferation 3. Wound healing 4. Cell migration 5. Tube formation 6. Angiogenesis protein expression	1. HT safe for cells up to 160 µM 2. HT enhanced wound healing process 3. HT stimulated HUVEC migration 4. HT upregulated migration and adhesion related protein expression such as ROCK, MMP-2, Phospho-Src, Src, Phospho Erk1/2, Erk1/2, RhoA, Rac1 and Ras 5. HT enhanced tube formation 6. HT upregulated VEGF) receptor 2 7. eNOS, PI3-Kinase, m-TOR, AMPK and Akt	1. PI3K/AKT/mTor 2. Erk1/2	HT positively regulated vascular remodeling by promoting reendothelization and wound healing by activating PI3K/AKT/mTor pathways
11	Wang et al. (2018)	To assess the effect of HT on autophagosis of VAFs and its related signaling pathways	Cells VAFs Treatment HT (12.5, 25, 50, 100, 200 and 400 µM) for 1 h followed by induction of TNF-α (5 ng/ml) for 24 h	1. Cell viability 2. SIRT1 siRNA level 3. Autophagy related protein level 4. Inflammatory cytokines level	1. HT was shown no cytotoxicity up to 100 µM 2. HT upregulated conversion of LC3 I to LC3 II and the expression of LC3 mRNA in VAFs stimulated with TNF-α 3. HT increased protein level and mRNA expression of Beclin1 4. HT regulated the expression of SIRT1 5. HT and SIRT1 shown compatibility in molecular docking 6. HT activated Akt/mTOR signaling pathway 7. HT decreased TNF-α induced inflammatory cytokine IL-1β	1. SIRT1 2. Akt/mTOR	Hydroxytyrosol promoted autophagy of VAFs via SIRT1- signaling pathway and inhibited inflammatory cytokines in vascular inflammation pathophysiology
12	Cheng et al. (2017)	To study the potential of HT together with PEMFs on HUVECs proliferation	Cells HUVECs Treatment PEMFs at days 0, 1, 2, 3 or 4, or treated with HTY (0, 10, 30, 50, 100, 150 µM) at day 2, or treated with a combination on days 0, 1, 2 or 4	1. Cell viability 2. Cell migration 3. Cell apoptosis	1. HTY + PEMF increases cell proliferation 2. HTY + PEMF enhanced cell migration 3. HTY + PEMFs prevented apoptosis 4. HTY increases mRNA and protein level of Akt, mTOR and TGF-β, but not p53	1. Akt 2. mTOR 3. TGF-β	PEMFs and HTY enhanced endothelial migration and proliferation that could promote reendothelization in vascular remodeling
13	Kouka et al. (2017)	To examine antioxidant property of pure HT from EVOO phenolic fraction	Cells 1. EA. hy926 2. C2C12 Treatment HT (0–40 μg/ml)	1. Extraction of TPF from EVOO 2. Purification of HT from TPF 3. radical scavenging assay 4. Cell viability 5. Assessment of GSH and ROS levels	1. HT exhibited highest antioxidant DPPH 2. HT reduced ROS 3. HT increased GSH		HT found to have decreased ROS and increased GSH which possibly enhance endothelial proliferation and functioning
14	Kitsati et al. (2016)	To assess the potential of HT in rescuing cells from oxidative stress induced by H_2_O_2_	Cells Jurkat cells Treatment HT (0.05 and 0.1 mM) for 30 min	1. Comet assay 2. Labile iron level 3. H_2_O_2_ generation	1. HT inhibited H_2_O_2_ induced labile iron level 2. Hydroxytyrosol inhibits H_2_O_2_-induced and mitochondrial-mediated apoptosis 3. Hydroxytyrosol inhibits H_2_O_2_-induced apoptosis 4. inhibits H2O 5. HT inhibited phosphorylation and activation of the JNK and p38 MAPKs	1. JNK 2. p38 MAPKs	HT prevented cellular apoptosis by inactivating JNK and p38 MAPKs pathway
15	Zrelli et al. (2015)	To examine the action of hydroxytyrosol in the vascular wound healing mechanism	Cells PPAECs Treatment HT (10–100 μM) 0–24 h	1. Expression of HO-1 and Nrf2 2. Wound healing	1. HT inclined HO-1 mRNA and protein level 2. HT induced HO-1 expression supported by PI3K/Akt and ERK1/2 3. HT mediated Nrf2 expression and nuclear localization	1. PI3K/Akt 2. ERK1/2 3. Nrf2	HT enhanced wound healing process in endothelial through activating expression of HO-1 and Nrf2
16	Zrelli et al. (2011a)	To study the effect of HT in vascular smooth muscle cell VSMCs proliferation	Cells RVSMCs Treatment HT (10, 30, and 100 µM) with and without 20 ng/mL of PDGF	1. Cell migration 2. Cell viability 3. NO production 4. Akt phosphorylation	1. HT decreased the number of viable VSMCs either in the presence or not of PDGF 2. HT promotes VSMCs apoptosis 3. HT increased NO production 4. HT increased iNOS protein expression 5. HT dephosphorylate Akt 6. PP2A mediated HT induced Akt phosphorylation	1. Akt 2. PPA	HT prevents VSMCs apoptosis through NO production and Akt dephosphorylation via activation of PP2A
17	Zrelli et al. (2011b)	To assess the proliferation and protective effect of HT on oxidative injury induced VECs injury	Cells PPAECs Treatment HT (10–100 µM) for 24 h followed by 0–700 3M) of H_2_O_2_ for 24 h	1. Cell viability 2. Wound healing 3. HO-1 mRNA expression 4. phosphorylation of Akt, p38 MAPK, and ERK1/2 5. ROS production	1. HT enhanced cell proliferation 2. HT repaired wound healing 3. HT prevented H2O2-Induced cytotoxicity 4. HT-induced phosphorylation of Akt, p38 MAPK, and ERK1/2 5. HT accumulates Nrf2 in nucleus 6. HT reduced ROS generation 7. HT increased mRNA and protein level of HO-1	1. Akt 2. MAPK 3. ERK1/2 4. Nrf2	HT protects VECs from oxidative damage through activation of the PI3K/Akt and ERK1/2 pathways
18	Catalan et al. (2015)	To evaluate the effect of hydroxytyrosol and its plasma metabolites toward endothelial protection	Cells HAEC Treatment HT (1, 2, 5, and 10 µM) co-incubated with TNF -α (10 ng/ml) for 18 and 24 h	1. HT metabolites production 2. Adhesion molecules production 3. Chemokine protein production 4. Cytotoxicity	1. HT and HT metabolites reduced E-selectin, P-selectin, VCAM-1, and ICAM-1 2. HT metabolites only reduced MCP-1		HT and HT metabolites exhibited vascular protection by reducing endothelial inflammation cytokines
19	Terzuoli et al. (2020)	To investigate the HT-3Os effects on endothelial-to-mesenchymal transition (EndMT) in the inflamed endothelium	Cells 1. EC 2. HUVEC 3. HREC Treatment 1. IL-1β (10 ng/ml) with or without HT-3Os (10 μM, every 24 h for 7 days	1. Morphology evaluation 2. Immunomarkers detection 3. Cytoplasmic and nuclear protein detection 4. miRNA expression analysis 5. Cytotoxicity	1. HT-3Os reverses EndMT-phenotypic changes induced by IL-1β 2. HT-3Os restores let-7 miRNA expression and inhibits TGF-β signaling 3. HT-3Os upregulated CD31 in IL-1β induced HUVEC and HREC 4. HT-3Os decreased fibroblast markers as FN1 and VIM or SMCin IL-1β induced HUVEC and HREC) 5. HT-3Os upregulated NOTCH3 and MMP2 and MMP9	1. let-7 miRNA 2. MMP 2 3. MMP 9	HT-3Os halts EndMT process in inflamed EC, by increasing let-7 miRNA expression and preventing activation of TGF-β signaling

**TABLE 2:** *In vitro* and *in vivo* studies outcome.

1	García et al. (2017)	To study effects of Hydroxytyrosol in endothelial cell expressing extracellular matrix remodeling enzymes in inhibition of angiogenesis	Animal and Cells 1. Rats 2. BAECs Treatment *In vitro*-HT 0–800 nmol) and 1 mM of HT for 24 h cells *In vivo*-HT 31.2,62.5, 125 and 250 µm) for 48 hours	1. *Ex vivo* rat aortic ring assay 2. *In vivo* chorioallantoic membrane (CAM) assay 3. mRNAs for some extracellular matrix remodeling enzymes	1. HT reduced MMP-1 and MMP-2, uPA mRNA expression 2. HT inhibit *ex vivo* angiogenesis, yet endothelial outgrowing observed 3. HT prevented *in vivo* angiogenesis		HT decreased expression of extracellular matrix remodeling enzyme that could supress migration of smooth muscle cells
2	Catalán et al. (2018)	To study the potential of hydroxytyrosol (HT) and its plasmatic metabolites (HTmet) in enhancement of endothelial function	Animal and cells 1. Apolipoprotein E knockout mice 2. HAEC 3. Jurkat Treatment Invivo-10 mg/kg/day of HT derivatives for 12 weeks Invitro-cells (1, 2 and 5 µM) and TNF-α (10 ng/ml) for 24 h	1. VCAM-1, E-selectin, MCP-1, ICAM-1 expression 2. Human Phospho-MAPK Array 3. NF-B (p65) expression	1. Mice aortas stained less for E-selectin, MCP-1, and ICAM-1 2. HTmet reduced Jurkat T adhesion 3. HTmet decreased E-selectin and VCAM-1 mRNA expression in HAECs 4. HT and HTmet decreased CREB, ERK, JNK pan, JNK, p38δ, p70 S6 kinase	1. ERK 2. JNK 3. MAPK	HT and its metabolites shown to have endothelial protection potential which regulated by the MAPK pathway
3	Yaoa et al. (2019)	To examine the potential of hydroxytyrosol acetate on vascular endothelial inflammation mechanism	Animal and Cells 1. Specific Sirt6 knockout mice hypercholesteraemic 2. HUVECs Treatment Invivo- P-407 (0.5 g/kg), P-407 + HT (5, 10, 20 mg/kg), and P-407+HT-AC (5, 10, 20 mg/kg) groups Invitro-HT or HT-AC (25, 50, or 100 μmol/L) for 1 h, and then stimulated with TNF (10 ng/ml) for 8 h	1. Cell viability 2. SOD, MDA and ROS level 3. SIRT6 siRNA transfection 4. SIRT6 and PKM2 expression 5. HT-AC molecular docking	1. HT and HT-AC decreased TNF and IL1B in mice serum 2. HT and HT-AC decreased mRNA expression of Il-b, Il6 and Ccl2 and TNF 3. HT and HT-AC decreased mRNA expressions of IL1B, IL6 and CCL2 in HUVECs 4. HT-AC increased SOD while decreased MDA and ROS level in TNF- induced HUVECs 5. HT-AC decreased TNFRSF1A protein and mRNA in HUVECs 6. HT-AC upregulated SIRT6 protein and mRNA expression in mice 7. Molecular docking shown good compatibility between HT-AC and SIRT6 8. HT-AC decreased expression of PKM2 in mice and TNF-stimulate HUVECs	1. PKM2	HT and HT-AC exhibited protection against endothelial inflammation in mice and HUVECs cells by mediating PKM2 signaling pathway
4	Fuccelli et al. (2018)	To study the effect of HT in inflammatory markers Cyclooxygenase-2 (COX2) And tumor necrosis factor alfa (TNF-α) and oxidative stress reduction in *vivo* systematic inflammation model	Animal Balb/c mice Treatment 1. HT (40 and 80 mg/kg) 2. LPS induction (50 µg/mouse)	1. COX2 mRNA detection 2. TNF-a cytokine determination 3. DNA damage assessment 4. Antioxidant plasma power quantification	1. HT inhibits the COX2 gene expression 2. HT reduces the TNF-α cytokine secretion 3. HT improves the antioxidant power of plasma 4. HT prevents the DNA damage induced	1. COX2 2. TNF-α	HT inhbited LPS induced COX2 expression, TNF-α production and the DNA damage while enhance antioxidant potential of plasma in *vivo* model

Abbreviations: THP-1, human monocyte cell line; U937, Monocytic cell line; HUVECs, Human umbilical vein endothelial cells; HMEC-1, Human microvascular endothelial cell line; PBMC, Human peripheral blood mononuclear cells; PPAECs, Porcine pulmonary artery endothelial cells; BVSMC, Bovine Vascular smooth muscle cells; HMECs, Human microvascular endothelial cells; VAFs, vascular adventitial fibroblasts; HVECs, Human vascular endothelium cells; BAECs, Bovine aorta endothelial cells; HAECs, human aortic endothelial cells; EA, hy926-endothelial cells; C2C12, myoblasts cells; HREC, Human retinal endothelial cells; RVSMCs, Rat Vascular smooth muscle cells; PMA, phorbol myristate acetate; MMP, matrix metalloproteinase; ROS, Reactive oxygen species; COX-2, cyclooxygenase 2; NF-κβ, nuclear factor kappa-light-chain-enhancer of activated B cells; MCP-1, monocyte chemoattractant protein-1; ICAM-1, intercellular adhesion molecule-1; VCAM-1, vascular cell adhesion molecule-1; IL-1β, interleukin-1β; TNF-α, tumour necrosis factor-α; HMVECs-d-Ad, Human dermal microvascular endothelial cells; VEGF, Vascular endothelial growth factor; prostaglandin (PG)E2; protein kinase C (PKC); FOXO3a, forkhead transcription factor 3a; AMPK-AMP, activated protein kinase; Akt, protein kinase B; CORM-2, Carbon Monoxide-Releasing Molecule-2; PEMF, Pulsed electromagnetic fields; mTOR-mechanistic target of rapamycin; TGF-β1, Transforming growth factor; MAPK, mitogen-activated protein kinase; EndMT, Endothelial-to-mesenchymal transition; HT-3Os, plasma metabolite HT-3O sulfate; FGFR1, fibroblast growth factor receptor 1

### Quality Evaluation

Risk bias analysis was conducted using modified version of Office of Health Assessment and Translation (OHAT). Overall, twenty-one out of twenty-three studies showed low risk bias. Two *in vitro*, two *in vivo* and one *in vitro* and *ex vivo* studies showed low risk of bias when they fulfill the selection criteria and reported all outcomes. In contrast, two studies showed high substantial risk of bias due to insufficient sample number and unclear adverse event reporting. A summary of risk bias analysis presented in Table 3.

**TABLE 3:** Presentation of risk bias analysis.

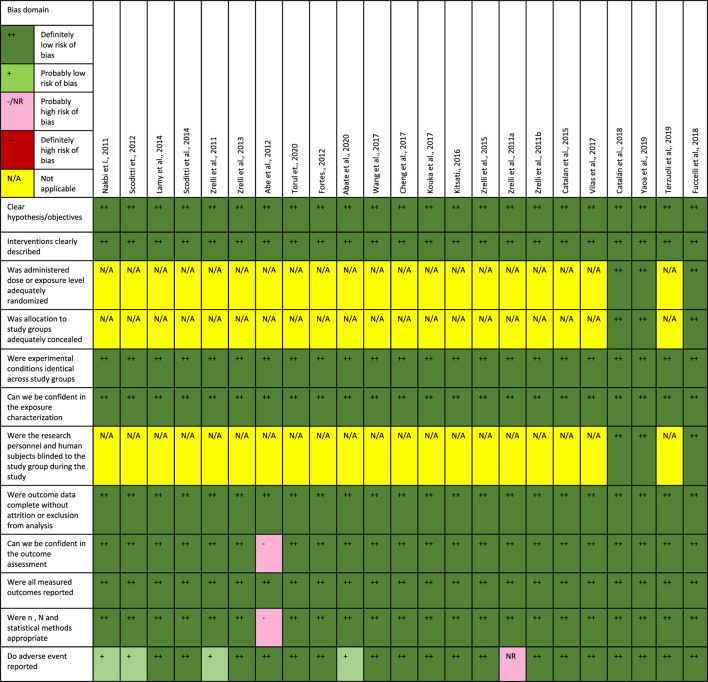

### HT Role as an Antioxidant

Antioxidant potential of HT comes from its chemical presence of hydroxyl (OH) groups in the ortho position. These OH groups are responsible in forming stable hydrogen bonds by scavenging reactive oxygen species (ROS) such as hydrogen peroxide (H_2_O_2_), superoxide ion (O_2_^−^), hydroxyl radical (OH^−^), and reactive nitrogen species (RNS) (Napolitano et al., 2010). HT regulates vascular homeostasis by balancing cellular oxidation stress and in addition to that, treatment with HT increase the production of nitric oxide (NO) which directly plays a crucial role in endothelial cells (ECs) functioning (Sandoo et al., 2010) such as inhibition of inflammation, cell adhesion (Reglero-Real et al., 2016), platelets interactions (Hamilos et al., 2018) as well as maintaining vessel tone (Norton and Popel, 2016).

Imbalance cellular redox reactions in ECs arise from vascular complications like thrombosis (Yang et al., 2017), intimal growth (Nedeljkovic et al., 2003), inflammation, and infarction (Pober and Sessa, 2007). These events are likely activate transcription factors which mediate the secretion of inflammatory factors and cells to the site of inflammation which eventually, delays the healing process (Martinon, 2010; Yang et al., 2017). Interestingly, Pi et al. showed that organic compound extracted from plant i.e. apocynin reduces endogenous ROS level in mice with carotid injury that subsequently suppressed the secretion of pro-inflammatory molecules and VSMC proliferation (Pi et al., 2013). Similarly, heart failure drug like simvastatin and Ivabradine reduces the generation of ROS in IH progression in hyperlipidaemic rabbits (Koniari et al., 2016).

These findings strongly support the correlation between oxidation machinery and the prevention of IH. From our database search, 9 studies reported that HT efficiently prevented ROS production (Nakbi et al., 2011; Zrelli et al., 2011a; Zrelli et al., 2011b; Scoditti et al., 2012; Zrelli et al., 2013; Kouka et al., 2017; Torul et al., 2020). HT was also reported to be able to phosphorylate endothelial nitric oxide synthase (eNOS) which increases nitric oxide (NO) synthesis that essentially needed for vascular integrity and protection (Tousoulis et al., 2011; Zhao et al., 2015; Loscalzo and Jin, 2010). This effect could potentially promote reendothelization in IH repair.

In addition to that, HT also protect cells from H_2_O_2_ induced cytotoxicity and apoptosis by decreasing superoxide release (Nakbi et al., 2011; Torul et al., 2020) while activating JNK and p38 MAPKs pathways (Kitsati et al., 2016). Interestingly, a particular study by Zrelli found that HT activate the AMPK-FOXO3 pathway by enhancing catalase activity to reduce oxidative stress (Zrelli et al., 2011b). Expression of FOXO3 appears to protect cells from oxidative injury by regulating the expression of the antioxidant enzyme such as catalase and peroxiredoxin (Hou et al., 2010; Olmos et al., 2009). Similarly, another set of studies stated AMPK directly activates FOXO3 transcriptional activity to provide cellular resistance toward oxidative stress (Greer et al., 2007; Li et al., 2009).

### HT Reduces Vascular Inflammatory Markers

Endothelial injury is a precursor for intimal hyperplasia (Garg and Hassid, 1989; de Vries and Quax, 2018). Inflammatory cytokines, chemokines, immune cells, and platelets are recruited to the site of injury to initiate repair mechanism which starts off with vascular inflammation and followed with healing process that are regulated by the immune system to maintain vascular health (D’Angelo et al., 2020). However, prolonged exposure to inflammatory molecules has a detrimental effect on vascular cells. Especially, during vascular injury, the secretion of ICAM-1 and MCP-1 attract platelet and leukocyte to the injured site. Gradually, the activated platelets trigger Thromboxane A2 and PDGF release which causes the VSMC to proliferate and migrate (Davies and Hagen, 1989; Huang et al., 2002). Thus, downregulating inflammatory factors and mediators potentially could prevent further progression of IH. Olive oil extracts have been shown to decrease the inflammatory activation in endothelial cells (Burja et al., 2019).

In ECs inflammation, nuclear factor-kappa B (NFκB) transcription factor regulates inflammatory mediators such as MCP-1, VCAM-1, ICAM-1, and E-selectin which recruits leukocytes, IL-6, and IL-8. (Pamukcu et al., 2011). From our systematic search, Scoditti et al. found that HT treatment decrease the expression of MMP-9, ICAM-1, IL-1b, TNF-α, and COX-2 by inactivating NF-κβ, PKCβ1, and PKCα in PMA activated human monocytes (Scoditti et al., 2014). Upon consumption, HT metabolized into glucuronide, sulfate methyl and methyl–sulphate conjugates (Kotronoulas et al., 2013; Rubió et al., 2014). It is crucial to test biological activity of HT metabolites together with HT assessing in vascular protection ability of HT. Catalan and colleagues synthesized physiological HT metabolites using Caco-2 cells. They reported that HT with its metabolites decrease inflammatory mediatorssuch as E-selectin, P-selectin, ICAM-1, and VCAM-1 but HT metabolite alone could only decrease MCP-1 level (Catalán et al., 2015). They further elucidate HT and HT metabolites potential in rat and endothelial cell model where they reported that HT and HT derivate supplemented aorta, stained less for E-selectin, MCP-1, and ICAM-1. Furthermore, they found that HT and HT metabolites provide endothelial protection through regulation of ERK, JNK, and MAPK interrelated pathways (Catalán et al., 2018). Moreover, Hydroxytyrosol acetate (HT-Ac), were also found to be able to suppress inflammatory response by upregulating SIRT-6 expression in hypercholesterolemic mice and TNF-α treated HUVECs. These studies shed light on the activation of TNFRSF1A and PKM2 pathways which are responsible for anti-inflammatory activity (Yao et al., 2019) thus proves HT inhibits inflammatory angiogenesis.

Inflammatory angiogenesis contribute immensely in the formation of tumor vasculature. Tumor angiogenesis produces new blood vessels from existing vessels to supply nutrients and oxygen to tumor cells (Aguilar-Cazares et al., 2019). HT successfully inhibited inflammatory angiogenesis in phorbol myristate acetate (PMA) stimulated endothelial cells through inhibition of proinflammatory enzyme cyclooxygenase (COX)-2 and matrix degrading enzymes matrix metalloproteinases (MMPs) which are proinflammatory mediators in cancer and atherosclerosis (Fortes et al., 2012; Scoditti et al., 2012).

### HT Enhances Re-endothelization

Re-endothelization is a prime event in IH repair. Delay in re-endothelization results in non-successful vascular interventions. Abate et al. reported that HT promote angiogenesis and wound healing in HUVECs cells via activating PI3K/AKT/mTOR pathways while upregulating the migration and adhesion-related protein expression (Abate et al., 2020). In another study, HT combined with pulsed electromagnetic field treatment, enhanced HUVECs migration and proliferation via regulation of Akt, mTOR, and TGF-β pathways (Cheng et al., 2017). Besides, two independent research by Zrelli et al. (2011b, 2015) demonstrates HT action of vascular healing through heme oxygenase-1 (HO-1) activation. High HO-1 expression protects cells from endothelial injury (Marcantoni et al., 2012; Kim et al., 2013). Additionally, they also reported that HT promotes vascular healing by stimulating the Nrf2 pathway which upregulates expression of HO-1 that is supported by PI3K, Akt, Erk ½. Lamy and colleagues, revealed that HT prevent endothelial apoptosis by suppressing ERK-1/2, SAPK and JNK pathways (Lamy et al., 2014).

### HT Inhibit VMSCs Proliferation and Migration

Proliferation and migration of VMSCs are huge contributors to intimal thickening. Naturally, VSMCs exist in both contractile and synthetic phenotypes which are responsible to maintain vascular homeostasis (Michel et al., 2012; Basatemur et al., 2019). Endothelial injury tends to trigger generation of inflammatory factors such as platelet-derived growth factor (PDGF), fibroblast growth factor (FGF), and transforming growth factor-beta (TGFβ), which accelerate the migration of VMSCs into the intima layer (Lindqvist et al., 2001). HT promote VMSCs apoptosis via the production of NO and subsequent inactivation of Akt mediated by PP2A pathway in PDGF induced rat VMSCs (Zrelli et al., 2011a).

Regulation of VSMCs proliferation determines by MAPKs family members such as c-Jun N terminal kinase (JNK), extracellular signal-regulated kinase ½ (ERK), and p38 (Xu et al., 2018). MAPK chains also promote PDGF-stimulated VSMCs migration in the vascular injury model (Zhan et al., 2003). In a study by Liu et al., sulphur dioxide prevented VSMCs proliferation by inactivating Erk/MAP kinase pathway (Liu et al., 2014). Therefore, HT successfully inhibit bovine VMSCs proliferation in the same manner by disrupting the cell cycle regulated by ERK ½ (Abe et al., 2012).

On another hand, Matrix Metalloproteinases (MMP) are crucial extracellular matrix (ECM) components in maintaining vessel integrity and angiogenesis (Raffetto and Khalil, 2008). Amongst the different type of MMPs, MMP-2 were shown to enhanced VMSCs migration by disrupting the ECM in an *in vitro* model (Belo et al., 2015). Therefore, HT's ability to inhibit MMP-2 expression (Fortes et al., 2012) could therefore suppress VSMCs migration. Just as important, expression of MMP-9 that breaks the barrier between VSMCs and ECs were found to be downregulated by HT treatment (Nakbi et al., 2011; Scoditti et al., 2012; Scoditti et al., 2014). Phenotype switching of VSMCs from contractile to synthetic, marks the beginning of VSMCs remodeling (Wadey et al., 2018). In a past study, Resveratrol stimulate differentiation of VSMCs and inhibit migration by activating SIRT1 and AMPK (Thompson et al., 2014). In the same way, HT regulate the expression of SIRT1 in TNF-α stimulated vascular adventitia fibroblast (VAFs). HT and SIRT1 were shown to have good compatibility (Wang et al., 2018). These findings thus support HT ability in prevention of excessive vascular remodeling.

## Discussion

Ethnopharmacology has been an ever-growing field especially in the discovery of new compound in treatments of various diseases. Linking our ancestor knowledge in medicinal plants and giving it a scientific prove are both exciting and beneficial in future medical treatment. The association of plant derived antioxidants, specifically Hydroxytyrosol (HT) with lower risk factor and mortality in cardiovascular disease patients that consume olives products are well recognized. HT were found to exerted cardioprotective and anti-atherosclerotic effects in a randomized, double-blinded, placebo-controlled, crossover trial that were performed for 20 weeks (Quirós-Fernández et al., 2019). However, until now HT has not been investigated in attenuating intimal hyperplasia (IH) which if found beneficial could change the treatment of CVD patients significantly.

Therefore, we compile studies that utilize HT in vascular remodeling and critically review the mechanism that were elucidated. Endothelial functioning and healing are a crucial point in preventing further progression of IH, as endothelial injury triggers migration of SMCs. HT antioxidant property provides an oxidative stress defense friendly environment that prevents endothelial dysfunction and apoptosis. This is facilitated by the activation of AMPK-FOXO3 (Zrelli et al., 2011b). The molecular action of HT downregulates NFκB pathway which improves NO production. HT also promote cellular survival from ROS induction (Torul et al., 2020). These series of evidence, allow us to proposed HT that could promote reendothelization in the site of endothelial injury.

Migration of smooth muscle cell (SMCs) is the direct causal effect following EC disruption in IH. Overall, direct effect of HT on SMCs were inhibition of proliferation and migration. HT inhibited SMCs migration and proliferation via blocking cell cycle regulated by ERK1/2 phosphorylation (Abate et al., 2012). Zrelli proved that NO production and Akt dephosphorylation could prevent VSMCs proliferation. He also reported these events triggered by activation of PP2A that leads to cell apoptosis (Zrelli et al., 2011a). Correspondingly, HT directly effect MMP 9 and MMP 2 reduction which indirectly inhibits migration of SMCs (Nakbi et al., 2011; Scoditti et al., 2012; Fortes et al., 2012; Scoditti et al., 2014).

With regards to dosage, up to 160 μM, HT promotes endothelial proliferation and functioning endothelium. HT efficiently reduced SMCs proliferation at a dosage of 100 µM. These findings strongly support our theory for the use HT as treatment for intimal hyperplasia where with further research, a perfect dosage that enables HT enhance reendothelization while inhibits SMCs migration. Therefore, we hope this evidence compilation will encourage researchers to investigate the use of HT in *ex vivo* intimal hyperplasia organ culture models in future.

## Conclusion

**FIGURE 2:**
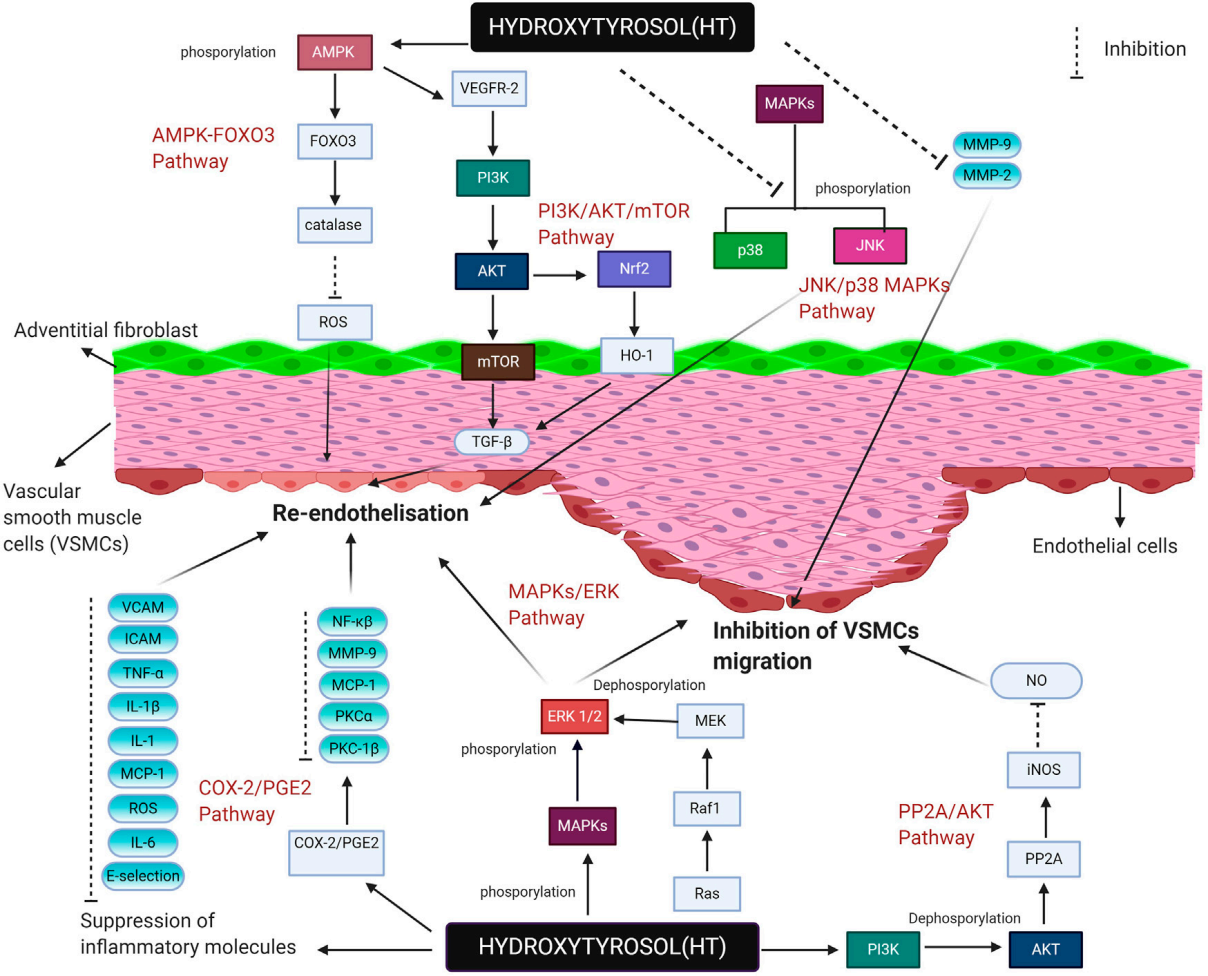
Hydroxytyrosol regulating key genes, inflammatory molecules and pathways in reendotelisation and inhibition of VSMCs

This systematic review collect evidences on molecular action of HT in the attenuation of IH in both *in vitro* and *in vivo* models. Supporting study on HT activity at the molecular level is presented in Tables 1 and 2 and further simplified in Figure 2. These consolidated findings uncovered the underlying pathways influenced by HT in IH suppression. HT promotes reendothelization by activating cell regulation pathways including AMPK/FOXO3, PI3K/AKT/mTOR and supressing VSMCs migration by disrupting cell cycle via inactivation of ERK1/2 and AKT. These findings can be further be applied in the treatment of IH by delivery of HT in future translational studies.

## Data Availability

The original contributions presented in the study are included in the article/Supplementary Material, further inquiries can be directed to the corresponding author.
